# A Low Cost VLSI Architecture for Spike Sorting Based on Feature Extraction with Peak Search

**DOI:** 10.3390/s16122084

**Published:** 2016-12-07

**Authors:** Yuan-Jyun Chang, Wen-Jyi Hwang, Chih-Chang Chen

**Affiliations:** Department of Computer Science and Information Engineering, National Taiwan Normal University, Taipei 117, Taiwan; 60347009s@ntnu.edu.tw (Y.-J.C.); 60447062s@ntnu.edu.tw (C.-C.C.)

**Keywords:** spike sorting, VLSI, brain machine interface

## Abstract

The goal of this paper is to present a novel VLSI architecture for spike sorting with high classification accuracy, low area costs and low power consumption. A novel feature extraction algorithm with low computational complexities is proposed for the design of the architecture. In the feature extraction algorithm, a spike is separated into two portions based on its peak value. The area of each portion is then used as a feature. The algorithm is simple to implement and less susceptible to noise interference. Based on the algorithm, a novel architecture capable of identifying peak values and computing spike areas concurrently is proposed. To further accelerate the computation, a spike can be divided into a number of segments for the local feature computation. The local features are subsequently merged with the global ones by a simple hardware circuit. The architecture can also be easily operated in conjunction with the circuits for commonly-used spike detection algorithms, such as the Non-linear Energy Operator (NEO). The architecture has been implemented by an Application-Specific Integrated Circuit (ASIC) with 90-nm technology. Comparisons to the existing works show that the proposed architecture is well suited for real-time multi-channel spike detection and feature extraction requiring low hardware area costs, low power consumption and high classification accuracy.

## 1. Introduction

There is an increasing demand in data-acquisition systems for neurophysiology to record simultaneously from many channels over long time periods [1]. These experiments accumulate large amounts of data, which would be processed by spike sorting systems for analyzing the activities of neurons. A typical spike sorting system [2,3] usually involves complicated feature extraction and spike classification operations for separating spikes from background noise and clustering the detected spikes. A large amount of spike trains would impose heavy computational load for a software spike sorting system, resulting in a long processing time.

One approach to reduce the computation time is to implement a spike sorting system by hardware. Hardware systems offering dedicated circuits substantially outperform their software counterparts in terms of computational performance. Hardware solutions are beneficial for neurophysiological signal recordings and analysis where real-time computing is crucial. There are two hardware approaches: Field Programmable Gate Array (FPGA) [4] and Application-Specific Integrated Circuit (ASIC) [5]. Some FPGA circuits [6,7] possess high area complexities and/or power consumption, which may be suited only for offline processing. On the contrary, ASIC architectures may have lower area costs and power dissipation. Many ASIC-based spike sorting implementations are then proposed for in vivo applications, where area- and power-efficient design is desired.

Many ASIC architectures based on Principal Component Analysis (PCA) [8,9,10] have been proposed for hardware spike sorting. Although they are effective for feature extraction, the inherent complexities for the computations of the covariance matrix and eigenvalue decomposition in the PCA algorithm may impose high hardware and power costs. The PCA variants, such as the Generalized Hebbian Algorithm (GHA) [11], are able to reduce the hardware costs by lifting the requirements for covariance matrix computation. In the GHA, the principal components are updated incrementally based on a set of training data. Nevertheless, its iterative training procedure may still be a bottleneck for online applications.

Alternatives to PCA and GHA include techniques such as discrete derivatives [12,13], integral transform [14] and zero-crossing [15] for feature extraction. The techniques feature low computational costs without additional training procedures. Nevertheless, some algorithms may be prone to noises due to the simple feature extraction procedure without taking noise into consideration. In addition, some of the algorithms have not been implemented by hardware. The effectiveness of the algorithms for ASIC implementation as compared with other techniques may still need to be evaluated.

The objective of this paper is to present a novel ASIC implementation for spike sorting featuring high classification accuracy, low area costs and low power consumption. The architecture is based on a novel feature extraction algorithm with low computational complexities. In the feature extraction algorithm, the location of the global minimum (or maximum) of a spike is first identified. Based on the location, the spike is then separated into two portions. The area of each portion is then used as a feature. Similar to the algorithms in [12,13,14,15], the proposed algorithm is simple to implement. In addition, it may be less susceptible to noise interference. Observe that the variations in the location of the global minimum (or maximum) of a spike due to noises may be small provided that the other local minimal (or maximal) values are significantly larger (or smaller) than the global one. This may be the case for some spike waveforms. The algorithm may then provide high immunity to noise corruption. Furthermore, the area of each position may be divided by the distance between the global maximum and minimum to enhance the classification accuracy.

A novel VLSI architecture is also presented for the novel feature extraction algorithm. In the architecture, the search for the global minimum and the computation of the area of portions separated by the minimum value are carried out concurrently. This is beneficial for pipelining operation for enhancing the throughput of the feature extraction. To further expedite the process, the local peak search and area computation over smaller segments of the spike can be carried out first. The local peak values and areas are subsequently merged with the global ones by a simple hardware circuit. In addition, due to its simplicity, the architecture can be operated in conjunction with other spike detection circuits. In this work, the circuit based on the Non-linear Energy Operator (NEO) [16] is employed for the multi-channel spike detection. To minimize the power consumption and area costs of the circuits, all of the channels share the same core for spike detection and feature extraction operations. A Clock Gating (CG) technique [17] is also employed to supply the system clock only to the active components of the circuit. A number of ASIC implementations are presented to demonstrate the effectiveness of the proposed architecture. Experimental results reveal that the proposed architecture is an effective alternative for in vivo multi-channel spike sorting with high classification accuracy, low power dissipation and low hardware area costs.

The remaining parts of this paper are organized as follows. The proposed feature extraction algorithm and the corresponding VLSI architecture are presented in Section 2. The architecture of the multi-channel spike sorting system supporting both the NEO and the proposed algorithm is presented in Section 3. Section 4 then evaluates the performance of the spike sorting system. Finally, Section 5 includes some concluding remarks.

## 2. The Proposed Feature Extraction Algorithm and Architecture

### 2.1. The Algorithm

#### 2.1.1. The Proposed Algorithm for the Feature Extraction of Spikes

This section presents a novel algorithm for the feature extraction of spikes. Consider a spike x with length *N*, where $x_{i},i=1,\ldots ,N,$ is the *i*-th sample of x. Let $i_{\min}$ and $i_{\max}$ be the index of the global minimum and maximum of x, respectively. That is,

(1) $$i_{\min}=\underset{1\leq i\leq N}{\mathrm{argmin}}x_{i},\;\;\;i_{\max}=\underset{1\leq i\leq N}{\mathrm{argmax}}x_{i}.$$

The index $i_{\min}$ (or $i_{\max}$) is used to separate the sample indices of spike x into two intervals $\left[1,i_{\min}\right]$ and $\left[i_{\min}+1,N\right]$. Let $a_{1}$ and $a_{2}$ be the two features based on $i_{\min}$. They are computed by:

(2) $$\begin{array}{ccc} a_{1} & = & \sum_{i=1}^{i_{\min}}\left(x_{i}-x_{i_{\min}}\right), \end{array}$$

(3) $$\begin{array}{ccc} a_{2} & = & \sum_{i=i_{\min}+1}^{N}\left(x_{i}-x_{i_{\min}}\right). \end{array}$$

The $a_{1}$ and $a_{2}$ can then be viewed as the area of the spike in the two intervals with $x_{i_{\min}}$ as the reference level. Alternatively, $x_{i_{\max}}$ can be used as the reference level. In this case, the separation of x is based on $i_{\max}$, and the corresponding features, denoted by $b_{1}$ and $b_{2}$, are given by:

(4) $$\begin{array}{ccc} b_{1} & = & \sum_{i=1}^{i_{\max}}\left(x_{i_{\max}}-x_{i}\right), \end{array}$$

(5) $$\begin{array}{ccc} b_{2} & = & \sum_{i=i_{\max}+1}^{N}\left(x_{i_{\max}}-x_{i}\right). \end{array}$$

In addition to the areas, the difference between $i_{\min}$ and $i_{\max}$ could be a feature beneficial for spike sorting. One way to incorporate the feature is based on the division as shown below.

(6) $$f_{i}=\left\{\begin{array}{cc} a_{i}/\left(i_{\min}-i_{\max}\right) & \mathrm{when}\;x_{i_{\min}}\;\mathrm{is}\;\mathrm{the}\;\mathrm{reference},\\ b_{i}/\left(i_{\min}-i_{\max}\right) & \mathrm{when}\;x_{i_{\max}}\;\mathrm{is}\;\mathrm{the}\;\mathrm{reference}, \end{array}\right.$$

where $f_{i},i=1,2,$ are the final features produced by the proposed algorithm.

**Figure 1:**
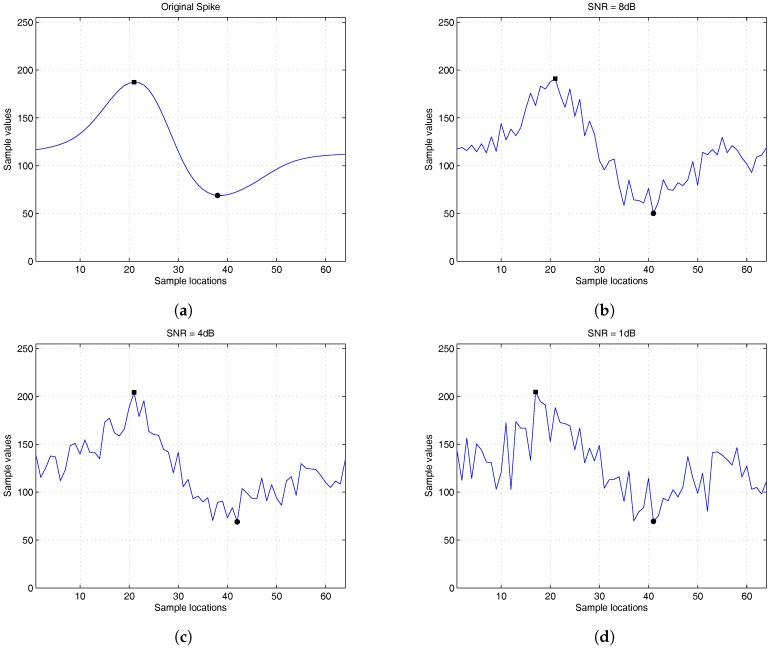
The locations and values of peaks of spikes corrupted by background noises with different SNR levels, where the black square and circle markers represent the maximum and minimum of spikes, respectively. (**a**) The original spike; (**b**) SNR = 8 dB; (**c**) SNR = 4 dB; (**d**) SNR = 1 dB.

An advantage of the proposed algorithm is that the feature vectors extracted by the algorithm may not be susceptible to noise interference. A contributing factor to the noise robustness is that the variations of the locations of peaks (i.e., $i_{\min}$ or $i_{\max}$) may not be high even for large background noises. For example, Figure 1 shows the locations and values of peaks of spikes corrupted by background noises with different SNR levels. Each spike contains 64 samples (i.e., N=64). The spike data for the figure is obtained from the simulator developed in [18]. It can be observed from Figure 1 that the location variations are small for all of the SNR levels considered in the figure. In particular, when SNR = 1 dB, the location of the minimum value is 41, as shown in Figure 1d. From Figure 1a, we see that the true location of the minimum value is 38. Because the length of spikes is 64, the variation is only 4.68%. This may be beneficial for maintaining low variations of $f_{i}$ in Equation (6) in the presence of noise.

**Figure 2:**
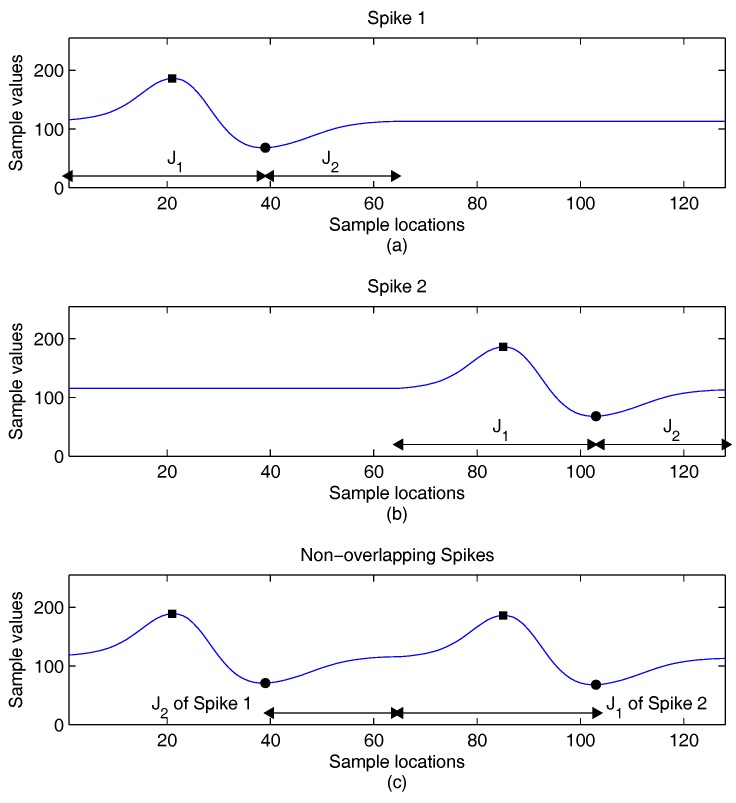
An example of two non-overlapping spikes for feature extraction. (**a**) Original Spike 1; (**b**) original Spike 2; (**c**) resulting waveform after the combination of the two spikes.

**Figure 3:**
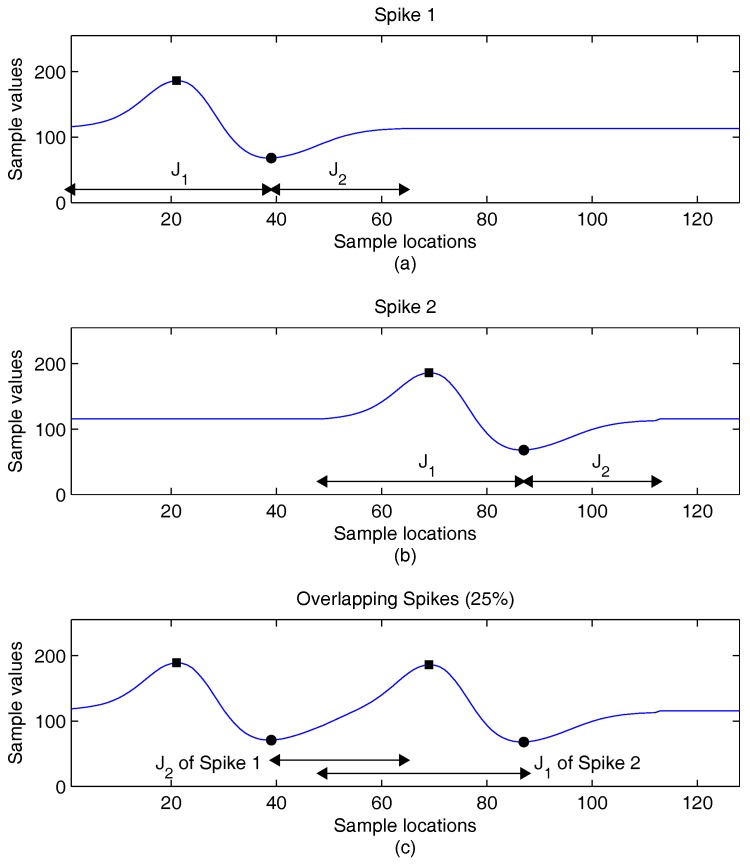
An example of two spikes overlapping by 25% for feature extraction. (**a**) Original Spike 1; (**b**) original Spike 2; (**c**) resulting waveform after the combination of the two spikes.

**Figure 4:**
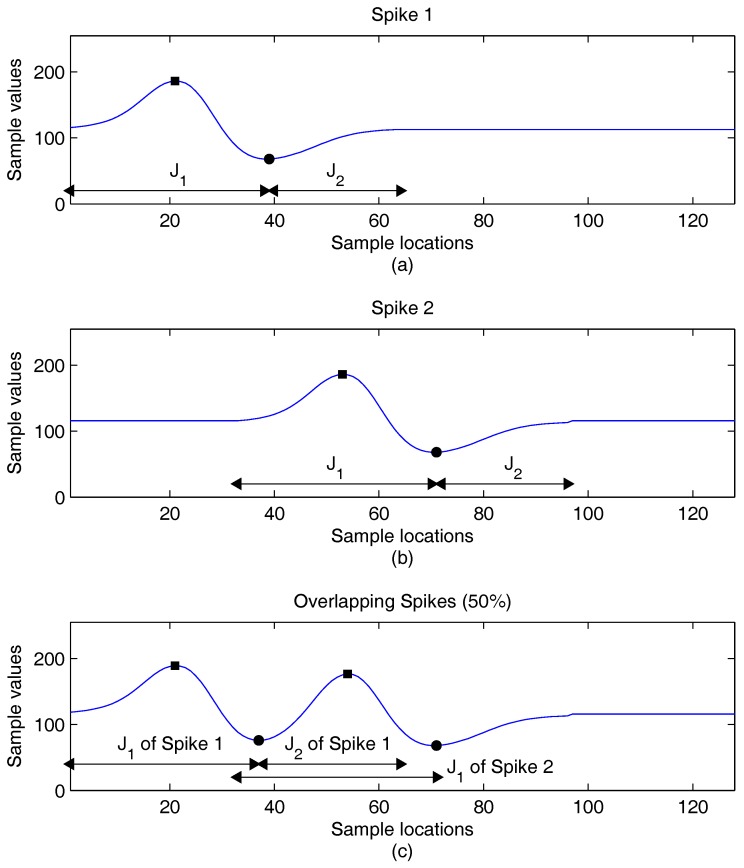
An example of two spikes overlapping by 50% for feature extraction. (**a**) Original Spike 1; (**b**) original Spike 2; (**c**) resulting waveform after the combination of the two spikes.

The variations of the locations of peaks may also be small when a spike is overlapped by another one. Examples of spikes with different degrees of overlapping are revealed in Figure 2, Figure 3 and Figure 4. To facilitate the observation, there is no background noise corruption in the spikes. In the examples, the location $i_{\min}$ of a spike is used to split the indices of spike samples into two intervals $\left[1,i_{\min}\right]$ and $\left[i_{\min}+1,N\right]$. For the sake of simplicity, we let $J_{1}=\left[1,i_{\min}\right]$ and $J_{2}=\left[i_{\min}+1,N\right]$. In addition to the locations and values of peaks, the $J_{1}$ and $J_{2}$ are marked for each spike in the figures. This is beneficial for the observance of the impact on the extraction of feature vectors due to spike overlapping.

We first consider the example without spike overlapping, as shown in Figure 2. There are two spikes in the example. The first and the second spike, denoted by Spike 1 and Spike 2, are located in the first 64 samples and final 64 samples in Figure 2a,b, respectively. Therefore, after the combination, the waveform of each individual waveform remains unaltered, as revealed in Figure 2c. That is, the peak locations stay the same, and the $J_{2}$ of Spike 1 is not overlapped with the $J_{1}$ of Spike 2. The feature vectors extracted from these two spikes will not be changed after the combination.

In the next example, Spike 2 is shifted leftward by 16 samples. Because the length of each spike is 64 samples, the Spike 1 and Spike 2 are overlapping by 25%, as shown in Figure 3. In this case, the combination of these two spikes introduces a slight distortion to each individual spike, as revealed in Figure 3c. Nevertheless, it can be observed that the variations in peak locations are small for each spike. Therefore, $J_{1}$ and $J_{2}$ can still be identified accurately for each spike. We can also see from Figure 3c that the $J_{2}$ of Spike 1 is overlapped with the $J_{1}$ of Spike 2. That is, one of the two areas separated by the $i_{\min}$ of Spike 1 also belongs to Spike 2, and vice versa. The $a_{2}$ of Spike 1 and $a_{1}$ of Spike 2 may then not be accurately extracted. However, because the remaining intervals (i.e., $J_{1}$ of Spike 1 and $J_{2}$ of Spike 2) are still non-overlapping intervals, the $a_{1}$ of Spike 1 and $a_{2}$ of Spike 2 can be computed accurately. The feature vectors may still be useful for subsequent spike classification.

Figure 4 shows the third example, where Spike 1 and Spike 2 are overlapping by 50%. We can see from Figure 4 that the peak location variations are still small even for this case. This is helpful for the successful identification of $J_{1}$ and $J_{2}$ for each spike. Due to the large area overlapping, the distortion of spike waveforms may be visible. This is particularly true for Spike 1 by comparing the waveform of the first 64 samples in Figure 4a with that of Figure 4c. This can also be further confirmed by observing from Figure 4c that both the $J_{1}$ and $J_{2}$ of Spike 1 are overlapped with both $J_{1}$ of Spike 2. The correct classification of Spike 1 may then be difficult. Nevertheless, the successful classification of Spike 2 may still be possible because its $J_{2}$ is not overlapped with the intervals of Spike 1, and accurate extraction of $a_{2}$ and subsequently classification for Spike 2 may still be successful.

Finally, when Spike 1 and Spike 2 are overlapping by more than 50%, large distortion of spike waveforms may be observed. The deviations of peak locations from their original ones before spike combinations may then be large, resulting in inaccurate identification of $J_{1}$ and $J_{2}$. In addition, larger overlapping of $J_{1}$ and $J_{2}$ of both spikes is possible. Consequently, accurate extraction of $a_{1}$ and $a_{2}$ of Spikes 1 and 2 may become more difficult in this case.

In summary, based on the results provided by the above examples, we see that the proposed algorithm may be robust to noise interference. The locations of peaks may not be susceptible to background noises and spike overlapping. The variations may be small even when the SNR = 1 dB and/or the degree of spike overlapping is up to 50%. The proposed algorithm is based on the locations of peaks for the computation of feature vectors. The robustness of the peak locations observed from the examples is then beneficial for providing useful insight into the effectiveness of the proposed algorithm.

#### 2.1.2. Operations of the Proposed Architecture Based on the Algorithm

From Equation (6), we see that the computation of $f_{i}$ is dependent of the selection of $x_{i_{min}}$ or $x_{i_{max}}$ as the reference. For the sake of simplicity, only the case where $x_{i_{min}}$ is the reference is considered for the hardware implementation. The hardware design for the other case can be followed in a similar fashion.

One direct approach for the hardware implementation of the proposed algorithm is to first identify $i_{\min}$ and $i_{\max}$ from the spike x. The computation of $a_{i}$ and $f_{i}$ is then followed. Although the approach is simple, it is necessary to store the spike x. This is because the spike x is first used for finding $i_{\min}$ and $i_{\max}$, and then, it is re-used again for computing $a_{i},i=1,2.$ As a consequence, the latency of the circuit may be long. In addition, the circuit may need a buffer to store x. When the dimension *N* of x is large, the area costs may be high.

The proposed architecture is able to alleviate the drawbacks stated above. It concurrently computes $i_{\min}$, $i_{\max}$ and $a_{i},f_{i},i=1,2.$ To carry out the concurrent operation, a novel incremental approach is proposed. Before presenting the approach, we first note that $a_{i},\;i=1,2,$ in Equations (2) and (3) can be rewritten as:

(7) $$\begin{array}{ccc} a_{1} & = & \left(\sum_{i=1}^{i_{\min}}x_{i}\right)-i_{\min}x_{i_{\min}}, \end{array}$$

(8) $$\begin{array}{ccc} a_{2} & = & \left(\sum_{i=i_{\min}+1}^{N}x_{i}\right)-\left(N-i_{\min}\right)x_{i_{\min}}. \end{array}$$

Both $a_{1}$ and $a_{2}$ can be computed after all of the *N* samples of x are available. In the case that only the first *j* samples of x are available, we define:

(9) $$p\left(j\right)=\underset{1\leq i\leq j}{\mathrm{argmin}}x_{i},\;\;\;q\left(j\right)=\underset{1\leq i\leq j}{\mathrm{argmax}}x_{i}.$$

as the current $i_{\min}$ and $i_{\max}$ up to the *j*-th sample of x, respectively. Based on p(j), the incremental versions of $a_{1}$ and $a_{2}$, denoted by $a_{1}\left(j\right)$ and $a_{2}\left(j\right)$, are defined as:

(10) $$\begin{array}{ccc} a_{1}\left(j\right) & = & S_{1}\left(j\right)-p\left(j\right)x_{p(j)}, \end{array}$$

(11) $$\begin{array}{ccc} a_{2}\left(j\right) & = & S_{2}\left(j\right)-\left(N-p\left(j\right)\right)x_{p(j)}, \end{array}$$

where:

(12) $$S_{1}\left(j\right)=\sum_{i=1}^{p(j)}x_{i},\;\;\;S_{2}\left(j\right)=\sum_{i=p(j)+1}^{j}x_{i}.$$

Clearly, when j=N, it follows from Equations (1) and (9) that $p\left(N\right)=i_{\min}$. Therefore, $a_{1}\left(N\right)=a_{1}$ and $a_{2}\left(N\right)=a_{2}$. Based on $a_{i}\left(j\right),i=1,2$, we then define $f_{i}\left(j\right),i=1,2,$ as:

(13) $$f_{i}\left(j\right)=a_{i}\left(j\right)/\left(p\left(j\right)-q\left(j\right)\right).$$

The $f_{i}\left(j\right)$ can also be viewed as the incremental version of $f_{i},i=1,2.$ From Equations (6) and (13), it can also be easily shown that $f_{i}\left(N\right)=f_{i}$ when $x_{i_{\min}}$ is used as the reference.

It is interesting to note that the computation of $S_{i}\left(j\right),i=1,2,$ can be carried out recursively from their predecessors $S_{i}\left(j-1\right),i=1,2.$ To explore the recursive relationship, two cases are considered separately. The first case is p(j)=p(j−1). This implies that the current $i_{\min}$ remains the same after the new sample $x_{j}$ has arrived. In this case,

(14) $$\begin{array}{ccc} S_{1}{\left(j\right)\;\;|}_{p(j)=p(j-1)} & = & \sum_{i=1}^{p(j)}x_{i}=\sum_{i=1}^{p(j-1)}x_{i}=S_{1}\left(j-1\right), \end{array}$$

(15) $$\begin{array}{ccc} S_{2}{\left(j\right)\;\;|}_{p(j)=p(j-1)} & = & \sum_{i=p(j)+1}^{j}x_{i}=\left(\sum_{i=p(j-1)+1}^{j-1}x_{i}\right)+x_{j}=S_{2}\left(j-1\right)+x_{j}. \end{array}$$

In the second case, p(j)≠p(j−1). This occurs only when $x_{j}=\min_{1\leq i\leq j}x_{i}$. Therefore, in this case, the current $i_{\min}$ is updated as p(j)=j. It then follows that:

(16) $$\begin{array}{ccc} S_{1}{\left(j\right)\;\;|}_{p(j)\neq p(j-1)} & = & \sum_{i=1}^{p(j)}x_{i}=\sum_{i=1}^{j}x_{i}=S_{1}\left(j-1\right)+S_{2}\left(j-1\right)+x_{j}, \end{array}$$

(17) $$\begin{array}{ccc} S_{2}{\left(j\right)\;\;|}_{p(j)\neq p(j-1)} & = & 0. \end{array}$$

All of the operations are based on the initial conditions $S_{1}\left(-1\right)=S_{2}\left(-1\right)=0$. We can observe from Equations (14)–(17) that $S_{1}\left(j\right)$ and $S_{2}\left(j\right)$ can always be obtained from $S_{i}\left(j-1\right),i=1,2,$ regardless of the variations of p(j). In addition to providing incremental computation, another important advantage is that it is not necessary to reuse $x_{j}$ for the computation of $S_{i}\left(k\right),i=1,2,$ for any k>j. This is beneficial for hardware design because it is not necessary to adopt a buffer for storing x for data re-use.

### 2.2. The Architecture

#### 2.2.1. Overview of the Proposed Architecture

**Figure 5:**
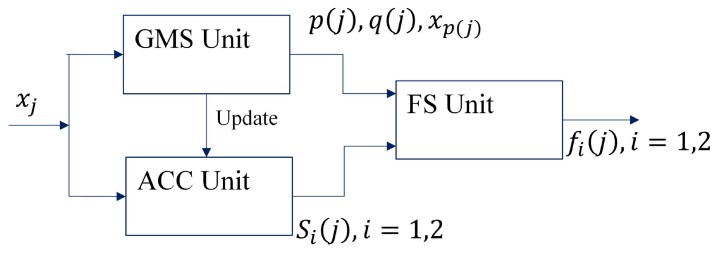
The proposed architecture for feature extraction.

Figure 5 shows the proposed architecture for feature extraction. As shown in Figure 5, it can be separated into three parts: the Global Minimum and maximum Search (GMS) unit, the ACCumulation (ACC) unit and the Feature Search (FS) unit. Based on an input sample $x_{j}$, the GMS unit computes p(j) and q(j), the current $i_{\min}$ and $i_{\max}$, respectively. The ACC unit then calculates $S_{i}\left(j\right),i=1,2$. Based on the results of GMS and ACC units, the FS unit produces the features $f_{i}\left(j\right),i=1,2.$

#### 2.2.2. GMS Unit, ACC Unit and FS Unit

**Figure 6:**
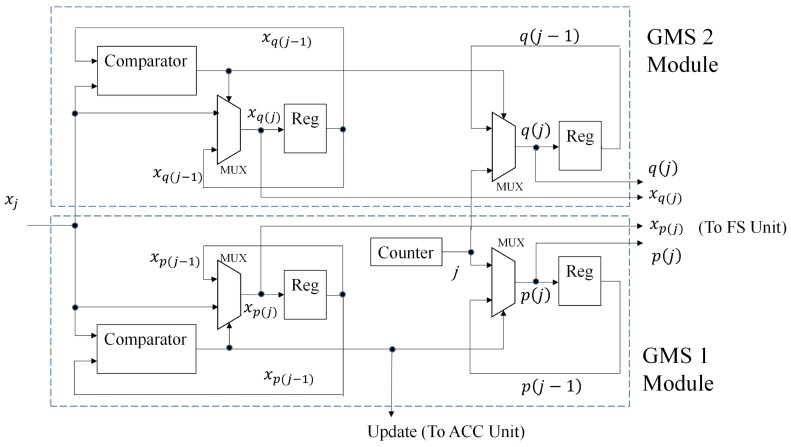
The architecture of the GMS unit.

The architecture of the GMS unit is depicted in Figure 6. The architecture updates and stores the current $i_{\min}$ and $i_{\max}$, as well as the current $x_{i_{\min}}$. It contained two modules: the GMS 1 module and the GMS 2 module. The goal of the GMS 1 module and GMS 2 module is to find the p(j) and q(j), respectively. Given an input sample $x_{j}$, in the GMS 1 module, the comparison between $x_{j}$ and $x_{p(j-1)}$ is carried out first. When $x_{j}>x_{p(j-1)}$, it follows from Equation (9) that p(j)=p(j−1). In this case, no updating occurs. Otherwise, we update p(j)=j, and $x_{p(j)}=x_{j}$. The module will also notify the occurrence of updating to the ACC unit. The GMS 2 module operates in a similar fashion for the updating of q(j), which occurs when $x_{j}>x_{q(j-1)}$.

**Figure 7:**
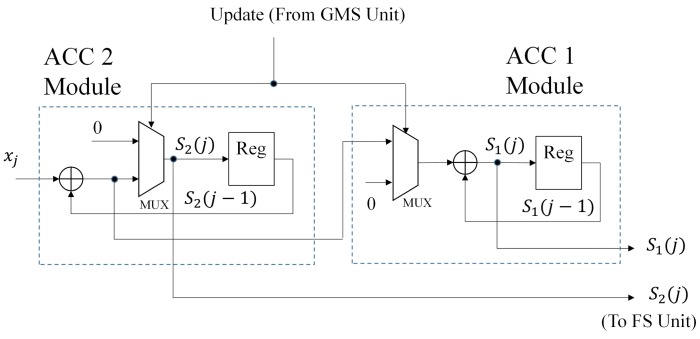
The architecture of the ACC unit.

There are two components in the ACC unit, termed the ACC 1 module and the ACC 2 module, respectively. Their goal is to compute $S_{1}\left(j\right)$ and $S_{2}\left(j\right)$. The operations of the ACC 1 and ACC 2 modules are based on Equations (14)–(17), respectively. As shown in Figure 7, each module contains a register, an adder and a multiplexer. As the input $x_{j}$ enters the proposed architecture, the current values held by the register of the ACC 1 and ACC 2 modules are $S_{1}\left(j-1\right)$ and $S_{2}\left(j-1\right)$, respectively. The input sample $x_{j}$ directly enters the ACC 2 module.

In the case that no updating operations are required (i.e., p(j)=p(j−1)), ACC 2 will add $x_{j}$ and $S_{2}\left(j-1\right)$ to compute $S_{2}\left(j\right)$ in accordance with Equation (15). In addition, from Equation (14), we see that $S_{1}\left(j-1\right)$ and $S_{1}\left(j\right)$ will be the same for ACC 1 module when no updating occurs. When p(j)≠p(j−1), ACC 2 will first compute $S_{2}\left(j-1\right)+x_{j}$ and then push this value to ACC 1. Meanwhile, based on Equation (17), the value of ACC 2 would then be reset to zero. Upon receiving the value $S_{2}\left(j-1\right)+x_{j}$ from ACC 2, ACC 1 adds the value to $S_{1}\left(j-1\right)$ according to Equation (16).

**Figure 8:**
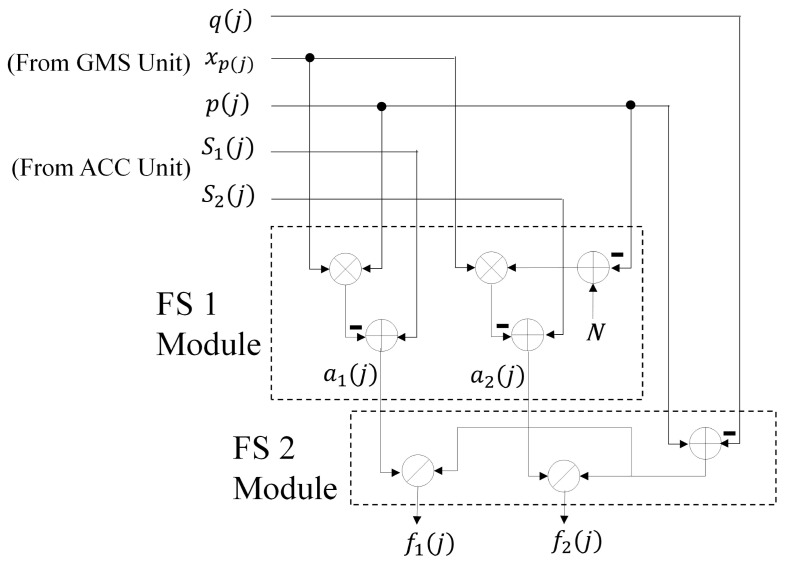
The architecture of the FS unit.

Figure 8 shows the architecture of the FS unit, which contains two components. The goal of the first component, termed the FS 1 module, is to compute $a_{1}\left(j\right)$ and $a_{2}\left(j\right)$ based on the results produced by the GMS unit and the ACC unit. The computation is carried out in accordance with Equations (10) and (11). Using the results produced by the FS 1 module, the second component of the FS unit, termed the FS 2 module, then computes the features $f_{1}\left(j\right)$ and $f_{2}\left(j\right)$ by Equation (13). As shown in Figure 8, both the FS 1 module and the FS 2 module mainly contain arithmetic operators for the hardware implementation of Equations (10), (11) and (13).

#### 2.2.3. Extension of the Proposed Architecture for Parallel Computation

The proposed architecture in its basic form operates on spikes one sample at a time. When the number of samples *N* in a spike is large, the latency of the computation may be long. One way to solve the problem is to separate a spike into non-overlapping segments and then operate on the segments concurrently. The computation time can then be reduced.

**Figure 9:**
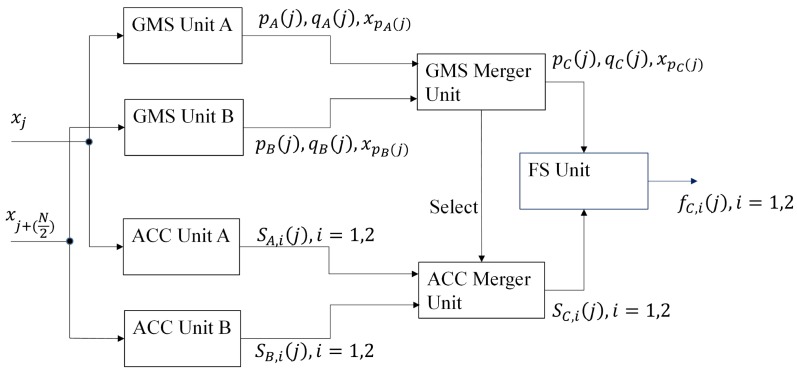
The proposed architecture for the concurrent operations with K=2.

Let *K* be the number of segments for the parallel computation, and *K* is a power of two. For the sake of simplicity, we first consider K=2. The computation for other cases of K>2 can be easily extended from this simple case. Figure 9 shows the proposed architecture for the concurrent operations with K=2. Because K=2, a spike will be separated into two segments. The samples $x_{j}$ with 1≤j≤(N/2) belong to the first segment and the others the second segment. There are two inputs: $x_{j}$ and $x_{j+N/2}$, which are the *j*-th sample of the first and the second segments, respectively. There are two GMS units and two ACC units. As shown in the figure, the units denoted by GMS Unit A and ACC Unit A are for the first segment. The others are for the second segment. Their computation results are then combined by the circuits termed the GMS merger unit and the ACC merger unit. Finally, the FS unit computes the final features based on the data provided by the GMS merger unit and the ACC merger unit.

To discuss the operations of the circuits, we first define sets A(j),B(j), and C(j) as:

(18) A(j)={i:1≤i≤j},B(j)={i:(N/2)+1≤i≤(N/2)+j},C(j)=A(j)∪B(j).

In addition,

(19) $$\begin{array}{ccc} p_{A}\left(j\right) & = & \underset{i\in A(j)}{\mathrm{argmin}}x_{i},\;\;\;p_{B}\left(j\right)=\underset{i\in B(j)}{\mathrm{argmin}}x_{i},\;\;\;p_{C}\left(j\right)=\underset{i\in C(j)}{\mathrm{argmin}}x_{i}. \end{array}$$

(20) $$\begin{array}{ccc} q_{A}\left(j\right) & = & \underset{i\in A(j)}{\mathrm{argmax}}x_{i},\;\;\;q_{B}\left(j\right)=\underset{i\in B(j)}{\mathrm{argmax}}x_{i},\;\;\;q_{C}\left(j\right)=\underset{i\in C(j)}{\mathrm{argmax}}x_{i}. \end{array}$$

Based on Equation (19), we further define:

(21) $$\begin{array}{ccc} S_{A,1}\left(j\right) & = & \sum_{i\in A\left(j\right),i\leq p_{A}\left(j\right)}x_{i},\;\;\;S_{A,2}\left(j\right)=\sum_{i\in A\left(j\right),i>p_{A}\left(j\right)}x_{i}. \end{array}$$

(22) $$\begin{array}{ccc} S_{B,1}\left(j\right) & = & \sum_{i\in B\left(j\right),i\leq p_{B}\left(j\right)}x_{i},\;\;\;S_{B,2}\left(j\right)=\sum_{i\in B\left(j\right),i>p_{B}\left(j\right)}x_{i}. \end{array}$$

(23) $$\begin{array}{ccc} S_{C,1}\left(j\right) & = & \sum_{i\in C\left(j\right),i\leq p_{C}\left(j\right)}x_{i},\;\;\;S_{C,2}\left(j\right)=\sum_{i\in C\left(j\right),i>p_{C}\left(j\right)}x_{i}. \end{array}$$

**Figure 10:**
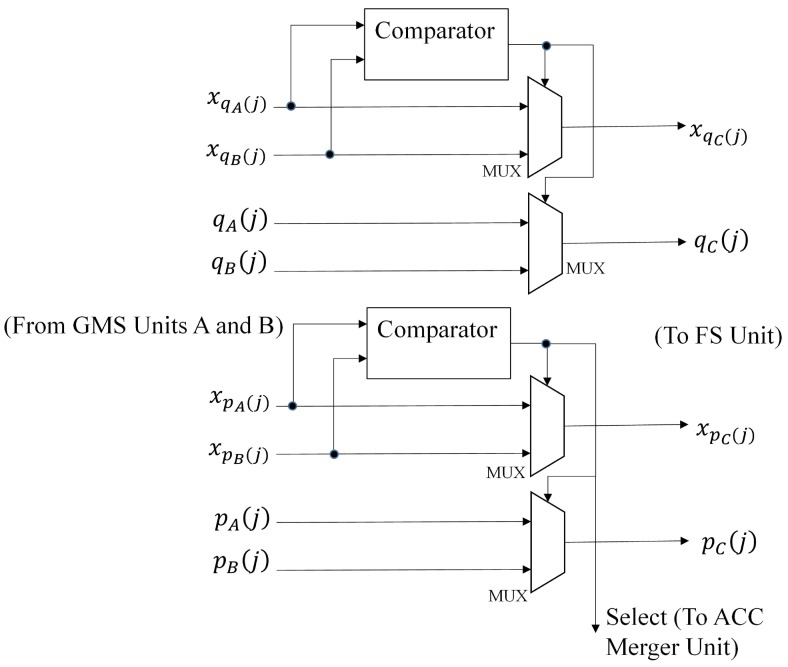
The architecture of the GMS merger unit.

**Figure 11:**
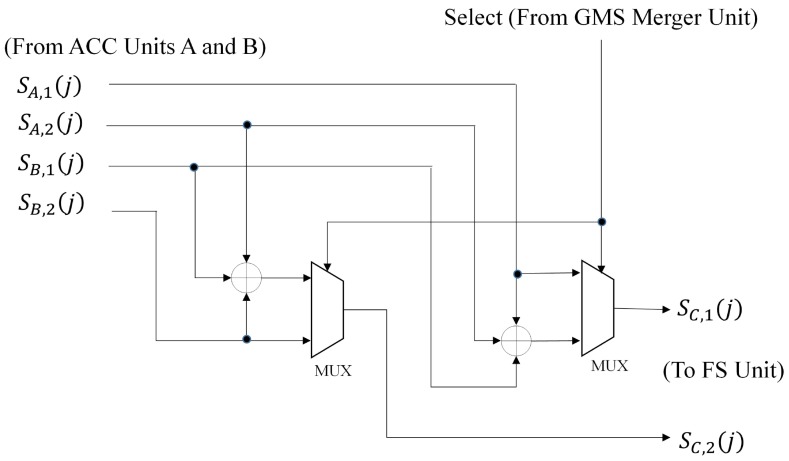
The architecture of the ACC merger unit.

The goal of GMS Units A and B is to compute $p_{A}\left(j\right),q_{A}\left(j\right)$ and $p_{B}\left(j\right),q_{B}\left(j\right)$, respectively. Their architecture is identical to that shown in Figure 6. The ACC Units A and B find $S_{A,1}\left(j\right),S_{A,2}\left(j\right)$ and $S_{B,1}\left(j\right),S_{B,2}\left(j\right)$, respectively. These units can be operated by the architecture in Figure 7. Based on $p_{A}\left(j\right),q_{A}\left(j\right)$ and $p_{B}\left(j\right),q_{B}\left(j\right)$, the GMS merger unit produces $p_{C}\left(j\right),q_{C}\left(j\right)$ (as well as $x_{p_{C}\left(j\right)}$). The ACC merger unit then computes $S_{C,1}\left(j\right),S_{C,2}\left(j\right)$. Figure 10 and Figure 11 shows the architectures of the GMS merger unit and the ACC merger unit, respectively.

It can be easily observed from Equations (19) and (20) that:

(24) $$p_{C}\left(j\right)=\left\{\begin{array}{cc} p_{A}\left(j\right) & \mathrm{if}\;x_{p_{A}\left(j\right)}\leq x_{p_{B}\left(j\right)}\\ p_{B}\left(j\right) & \mathrm{if}\;x_{p_{A}\left(j\right)}>x_{p_{B}\left(j\right)} \end{array}\right.$$

(25) $$q_{C}\left(j\right)=\left\{\begin{array}{cc} q_{A}\left(j\right) & \mathrm{if}\;x_{q_{A}\left(j\right)}\geq x_{q_{B}\left(j\right)}\\ q_{B}\left(j\right) & \mathrm{if}\;x_{q_{A}\left(j\right)}<x_{q_{B}\left(j\right)} \end{array}\right.$$

Therefore, as shown in Figure 10, the GMS merger unit carries out the comparison operations over $x_{p_{A}\left(j\right)}$ and $x_{p_{B}\left(j\right)}$ (or $x_{q_{A}\left(j\right)}$ and $x_{q_{B}\left(j\right)}$) for finding $p_{C}\left(j\right)$ (or $q_{C}\left(j\right)$). Only comparators and multiplexers are required for the operations. We can also derive from Equations (21)–(24) that when $p_{C}\left(j\right)=p_{A}\left(j\right)$,

(26) $$\begin{array}{ccc} S_{C,1}{\left(j\right)\;\;|}_{p_{C}\left(j\right)=p_{A}\left(j\right)} & = & S_{A,1}\left(j\right), \end{array}$$

(27) $$\begin{array}{ccc} S_{C,2}{\left(j\right)\;\;|}_{p_{C}\left(j\right)=p_{A}\left(j\right)} & = & S_{A,2}\left(j\right)+S_{B,1}\left(j\right)+S_{B,2}\left(j\right). \end{array}$$

On the other hand, when $p_{C}\left(j\right)=p_{B}\left(j\right)$,

(28) $$\begin{array}{ccc} S_{C,1}{\left(j\right)\;\;|}_{p_{C}\left(j\right)=p_{B}\left(j\right)} & = & S_{A,1}\left(j\right)+S_{A,2}\left(j\right)+S_{B,1}\left(j\right), \end{array}$$

(29) $$\begin{array}{ccc} S_{C,2}{\left(j\right)\;\;|}_{p_{C}\left(j\right)=p_{B}\left(j\right)} & = & S_{B,2}\left(j\right). \end{array}$$

The operations of the ACC merger unit then follow Equations (26)–(29). These operations can be carried out by only adders and multiplexers, as shown in Figure 11.

Based on the data produced by the GMS merger unit and ACC merger unit, the FC unit then computes $f_{C,1}\left(j\right)$ and $f_{C,2}\left(j\right)$, defined as:

(30) $$f_{C,i}\left(j\right)=a_{C,i}\left(j\right)/\left(p_{C}\left(j\right)-q_{C}\left(j\right)\right),$$

where:

(31) $$\begin{array}{ccc} a_{C,1}\left(j\right) & = & S_{C,1}\left(j\right)-p_{C}\left(j\right)x_{p_{C}\left(j\right)}, \end{array}$$

(32) $$\begin{array}{ccc} a_{C,2}\left(j\right) & = & S_{C,2}\left(j\right)-\left(N-p_{C}\left(j\right)\right)x_{p_{C}\left(j\right)}, \end{array}$$

The architecture of the FS unit for parallel computation is also identical to that of the basic circuit shown in Figure 8. It can be observed that, when j=N/2, C(N/2)={i:1≤i≤N}. Therefore, $a_{C,i}\left(N/2\right)=a_{i},i=1,2$. As a result, $f_{C,i}\left(N/2\right)=f_{i},i=1,2$.

The proposed architectures can also be viewed as trees. The basic architecture shown in Figure 5 is a unitary tree with a leaf node and a root node. The parallel computation circuit in Figure 9 for K=2 can be viewed as a simple binary tree with two leaf nodes, one intermediate node and one root node. Each leaf node contains a GMS unit and an ACC unit. Each intermediate node consists of a GMS merger unit and an ACC merger unit. The root node comprises the FS unit.

**Figure 12:**
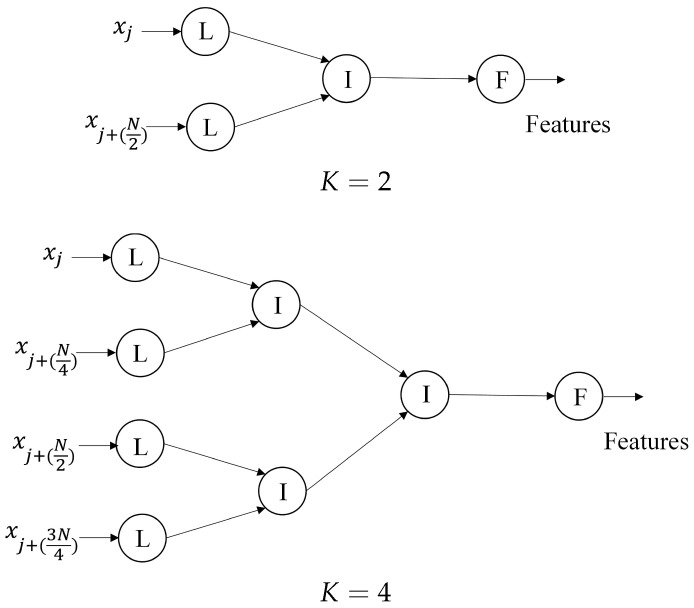
The binary tree representation of the proposed architecture for parallel computation with K=2 and 4. The nodes denoted by L, I and F are the leaf nodes, intermediate nodes and root nodes, respectively.

The parallel computation for a larger number of *K*, where *K* is a power of two, is a simple extension of K=2. In this case, the circuit can be viewed as a binary tree with *K* leaf nodes and K−1 intermediate nodes and a root node. There are $2+\log_{2}K$ layers for the parallel computation. The leaf nodes form the first layer. The intermediate nodes can be organized into the subsequent $\log_{2}K$ layers. The root node is the final layer of the circuit. Figure 12 shows the examples of the binary trees for K=2 and K=4.

The circuit with *K* a power of two operates by first separating a spike into *K* non-overlapping segments with length N/K. Each segment is assigned to a dedicated leaf node. Samples of each segment are delivered to the assigned leaf node one sample at a time. The data produced by the leaf nodes are then forwarded to the first layer of the intermediate nodes. Each layer would then receive data from its preceding layer and deliver the results to the next layer. The same process is repeated until the final layer is reached.

## 3. Proposed Architecture for Multi-Channel Feature Extraction

**Figure 13:**
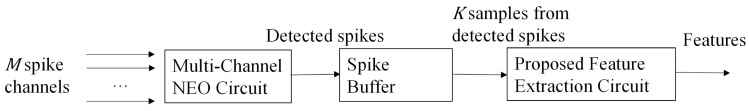
The architecture of a multi-channel spike sorting system based on the proposed feature extraction circuit.

The proposed architecture can be easily operated in conjunction with the multi-channel spike detection circuit for multi-channel feature extraction. Figure 13 shows the architecture of a multi-channel spike sorting system based on the proposed feature extraction circuit. As depicted in the figure, there are three components: spike detection circuit, spike buffer and the proposed feature extraction circuit.

The spike detection circuit is able to carry out the detection for multiple channels by the NEO algorithm. Let *M* be the number of channels for spike sorting. Assume all of the channels are sampled with the same sampling rate $r_{s}$. Let $T_{s}=1/r_{s}$ be the sampling period. All of the channels are assumed to be sampled and multiplexed by a mixed mode circuit using the round robin approach. The mixed-mode circuit then delivers the samples one at a time to the spike detection circuit. Therefore, the circuit receives *M* samples during a time interval of length $T_{s}$. Different samples received during the interval are from different channels.

The spike detection circuit can be separated into two portions: the NEO buffer and the NEO detection unit. The NEO buffer is a (M×N)-stage Serial-In-Serial-Out (SISO) shift register organized in a snake-like fashion. Each stage contains a sample of a spike train from a channel. It is therefore able to hold *N* consecutive samples of the spike trains from the *M* channels.

**Figure 14:**
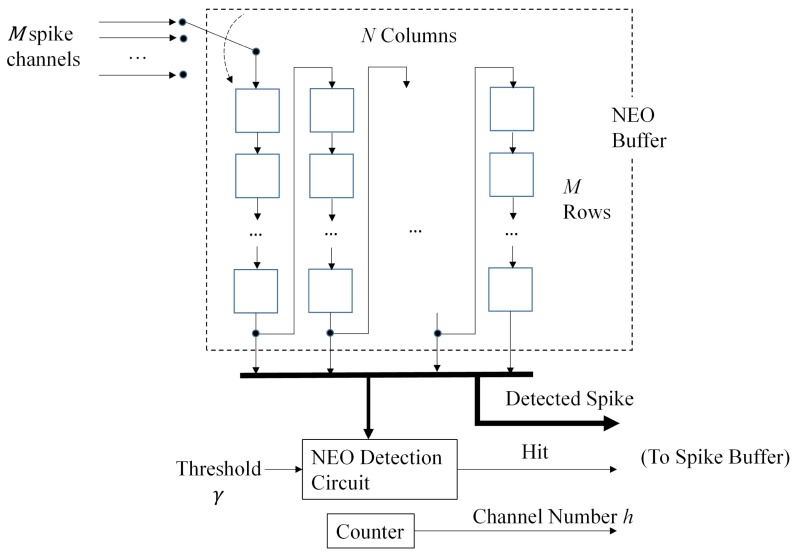
The architecture of the multi-channel NEO circuit.

The bottom row of the buffer provides *N* consecutive samples of the spike train from a channel (say, channel *h*). It can be seen from Figure 14 that the bottom row of the NEO buffer is used for both NEO detection and peak alignment. The NEO detection unit takes three consecutive samples of the bottom row to carry out the NEO computation. The computation result is then compared to a given threshold *γ*. If the result is larger than the threshold, a hit event is issued. In addition, the entire last row is regarded as a detected spike and is copied to the spike buffer for spike alignment.

The spike detection circuit is able to perform spike detection one channel at a time. After the spike detection of the channel *h* is completed in the current clock cycle, all of the spike samples already in the NEO buffer are shifted to the next stage, and a new sample from the next channel (selected in a round robin fashion) enters the first stage of the NEO buffer. This allows the spike detection for the next channel to be carried out in the next clock cycle.

**Figure 15:**
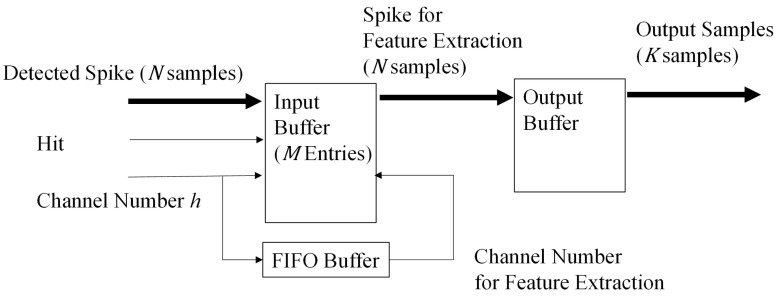
The architecture of the spike buffer.

The goal of the spike buffer is to hold the detected spikes produced by the spike detection circuit, carry out the alignment and deliver the detected spikes to the proposed feature extraction circuit. As depicted in Figure 15, there are two stages in the spike buffer: the input stage and the output stage. The input stage is an *N*-input *N*-output buffer holding the detected spikes provided by the spike detection circuit. The output stage is an *N*-input *K*-output buffer fetching data *N* samples at a time from the input buffer. The buffer then delivers data *K* samples at a time to the proposed feature extraction circuit.

The input stage contains *M* entries, where each entry *i* stores the detected spike for channel *i*. Given *N*, *K*, the maximum number of channels *M* can be evaluated. For any channel *h* in the circuit, a detected spike in that channel could be discarded when the spike is over-written in the spike buffer by the next detected spike from the same channel *h* before it can be further processed.

Recall from Section 2.2.3 that the proposed architecture supporting parallel computation is able to accept *K* samples at a time. Therefore, the proposed architecture is able to process a detected spike with *N* samples in N/K clock cycles. To find the maximum number of channels *M*, the worst case scenario is considered. In the scenario, the detected spikes from all of the *M* channels are stored in the spike buffer and are not processed by the feature extraction circuit yet. In addition, the feature extraction circuit is currently busy. Assume that the newest detected spike is from channel *h*. In this case, the spike buffer is able to accept another detected spike from channel *h* without discarding the old one only after the feature extraction circuit has processed *M* spikes. Because the latency for processing each detected spike is N/K clock cycles, it follows that the input buffer is able to accept another detected spike from channel *h* after MN/K clock cycles. Let *Q* be the minimum number of samples between the peak of successive spikes detected by the NEO circuit from the same channel. Assume that *Q* is the same for all of the channels. It then follows that a detected spike from channel *h* is not overwritten and discarded when:

(33) $$\frac{MNT_{c}}{K}\leq QT_{s},$$

where $T_{c}=1/r_{c}$ is the clock period and $r_{c}$ is the clock rate. It is interesting to know that the NEO circuit imposes an additional limit on the number of channels *M*. It is desired that the NEO circuit be able to receive one sample from each channel in a single sampling period $T_{s}$. Based on the round robin scheme for fetching samples for different channels, it is therefore necessary that:

(34) $$MT_{c}\leq T_{s}.$$

Combining Equations (33) and (34), we see that the maximum number of channels, denoted by $M_{\max}$, should then satisfy:

(35) $$M_{\max}\leq \min\left\{\frac{QT_{s}K}{NT_{c}},\frac{T_{s}}{T_{c}}\right\}.$$

## 4. Experimental Results

This section presents the performance of the proposed architecture. The area complexities are first considered. Five types of area costs are evaluated: the number of comparators, adders/subtractors, multipliers/dividers, registers and multiplexers. All of the costs are expressed in terms of the asymptotic function (i.e., the big *O* function). Table 1 shows the area complexities of the proposed feature extraction circuit. It can be observed from Table 1 that all of the area costs of the GMS units, ACC units, GMS merger units, ACC merger units and FS units are independent of the spike length *N* and the number of channels *M*. However, for the parallel computation, the number of comparators, adders/subtractors, registers and multiplexers of all of the leaf nodes will grow with the number of segments *K*. This is because the total number of leaf nodes is dependent on *K*, and each leaf node contains a GMS unit and an ACC unit.

Similarly, because the total number of intermediate nodes is dependent on *K* and each intermediate node contains a GMS merger unit and an ACC merger unit, the area complexities of comparators, adders/subtractors and multiplexers of all of the intermediate nodes are O(K). On the contrary, there is only one root node for parallel computation, and the root node consists of only the FS unit. Among the units in the proposed architecture, the FS unit is the only unit containing multipliers/dividers. Therefore, the number of multipliers/dividers is fixed, and is independent of *N*, *M* and *K*. Summarizing the discussions stated above, we conclude that the complexities of the total number of comparators, adders/subtractors, registers and multiplexers of the proposed feature extraction circuit with *K* segments are O(K). Moreover, the total number of multipliers/dividers is only O(1).

Based on Table 1, the overall area complexities of the proposed spike sorting circuit are summarized in Table 2. To facilitate the evaluation, the area complexities of the NEO circuit and spike buffer are also included in the table. For the NEO circuit, it is necessary to store spike trains from all of the *M* channels for detection. In the spike buffer, each channel needs to have its own memory unit to hold the detected spikes for the subsequent feature extraction. Therefore, it can be observed from Table 2 that the number of registers in both the NEO circuit and spike buffer are dependent on the spike length *N* and the number of channels *M*. The total number of registers of the proposed spike sorting circuit is then O(K+MN). In addition, because the NEO circuit has only a fixed number of adders and multipliers independent of *N* and *M*, the overall area complexities for the adders and multipliers in the spike sorting circuit are only O(K) and O(1), respectively.

We next evaluate the actual hardware resource consumption of the proposed circuit. For the remaining evaluations of this section, the dimension of spikes is N=64. The circuit implementation is carried out by ASIC with the Taiwan Semiconductor Manufacturing Company (TSMC) 90-nm technology and clock gating. The gate level design platform is the Synopsys Design Compiler. Table 3 shows the area (μm${}^{2}$) of the proposed circuit for different numbers of channels *M* and numbers of segments *K*. From Table 3, we see that the area of the proposed circuit grows with *M* and *K*, which is consistent with the results shown in Table 2. Table 4 shows the normalized area (μm${}^{2}$ per channel) of the proposed architecture. The normalized area is defined as the area of the circuit divided by the number of channels *M*. The normalized area can be viewed as the average area cost per channel. Note that all of the channels share the same computation cores in the NEO circuit (i.e., the NEO detection unit) and the proposed feature extraction circuit. Therefore, we can see from Table 4 that the average area per channel decreases as *M* increases. Consequently, because of the hardware resource sharing scheme, the proposed architecture shows a higher efficiency in area costs for a larger number of channels. Although the normalized area grows with *K* for a fixed *M*, the circuits with larger *K* have the advantages of lower latency for feature extraction. Therefore, as shown in Equation (35), the channel capacity $M_{\max}$ grows with *K*.

In addition to the area costs, the power consumption of the proposed architecture is also evaluated. Table 5 shows the normalized power dissipation (μW per channel) for different numbers of channels *M* with and without clock gating. The normalized power dissipation is defined as the total power consumption of the circuit divided by the number of channels *M*. In the experiment, the number of segment K=1, and the clock rate $r_{c}$ is 1 MHz. The power consumption is measured numerically by Synopsis Prime Time. When the number of channels *M* increases, because the normalized area decreases, we can see from Table 5 that the normalized power consumption also lowers for the circuit without clock gating. In addition, the clock gating is able to further reduce the power consumption by not supplying the clock signal to the inactive components. However, when *M* increases, the components of the proposed feature extraction circuit may become more busy because all of the *M* channels share the circuit. As a result, when *M* increases from 32–64, it can be observed from Table 5 that the power consumption with clock gating is not lowered. Nevertheless, the the circuit with clock gating is still able to achieve a 33% power reduction as compared with its counterpart without clock gating.

We also compare the proposed architecture with other ASIC implementations [8,9,10,11] for spike sorting in Table 6. It may be difficult to directly compare these architectures because they may be implemented by different technologies and may be based on different spike detection and/or feature extraction algorithms. Moreover, their operation clock rates may be different. Nevertheless, it can be observed from Table 6 that, as compared with the existing architectures, the proposed architecture provides effective area-power performance while supporting both spike detection and feature extraction functions.

Note that, among these existing architectures, the proposed architecture and the one in [11] are based on the same clock rate, technology and spike detection scheme. We can see from Table 6 that the normalized area and normalized power of the proposed architecture are only 25.40% (i.e., 21,127 vs. 83,159) and 25.44% (i.e., 20.03 vs. 78.719) of those of the architecture in [11], respectively. This is because the proposed feature extraction algorithm has significantly lower computational complexities than those of the GHA algorithm adopted by [11].

Although the proposed feature extraction algorithm has simple computation, it is effective for spike classification. Table 7, Table 8, Table 9 and Table 10 show the Classification Success Rate (CSR) of the Fuzzy C-Means (FCM) [19] algorithm by clustering the feature vectors produced by various feature extraction algorithms. The CSR is defined as the number of spikes that are correctly classified divided by the total number of spikes. The PCA, GHA and zero-crossing [15] algorithms considered in the tables are implemented by MATLAB with double precision floating numbers. The proposed algorithm is implemented by hardware with 10-bit finite precision.

To see the robustness of the proposed algorithm, different types of noise interference are included in the experiments: background noises, interfering neurons and overlapping spikes. They are considered separately to facilitate our observation. The spike trains for the experiments in Table 7, Table 8 and Table 9 are obtained from the simulator developed in [18]. It also gives access to the ground truth about the spiking activity in the recording for quantitative assessment.

Table 7 shows the CSRs of various feature extraction algorithms for background noises with different SNR levels. In the experiments, the number of interfering neurons is set to be zero. In addition, 20% of the spikes are overlapping. It can be observed from Table 7 that the CSRs of the proposed algorithm are only slightly degraded as the SNR values decrease. In addition, the proposed algorithm has CSR performance comparable to that of PCA and GHA for all of the SNR levels. Moreover, it outperforms the zero-crossing algorithm. When the SNR = 1 dB, the proposed algorithm is still able to achieve 96.47% CSR. As shown in Figure 1, the proposed algorithm is robust to background noise because the variations of peak locations due to background noise corruption are small. This leads to successful identification of intervals $J_{1}$ and $J_{2}$ for the computation of features $a_{1}$ and $a_{2}$.

The CSRs of various algorithms for different numbers of interfering neurons are revealed in Table 8. The interfering neurons are the nearby neurons whose spike times are correlated with the original spike times of the target neurons. We set SNR = 4 dB for the background noises. The percentage of the overlapping spikes for the experiments is 20%. From Table 8, we can see that only a small degradation is observed for the proposed algorithm as the number of interfering neurons grows. Its performance is also comparable to that of the PCA and GHA algorithms.

The influences of the overlapping spikes on CSRs are considered in Table 9. In the experiments, the SNR level of background noises is 8 dB. In addition, there are no interfering neurons, so that the the impact of overlapping spikes can be easily observed. It appears from Table 9 that the proposed algorithm is able to maintain CSRs above 95% even when the percentage of the overlapping spikes is 30%. Because the proposed algorithm may still be able to find the $J_{1}$ or $J_{2}$ correctly for the overlapping spikes as revealed in Figure 3 and Figure 4, successful feature extraction and classification may still be possible. As a result, the proposed algorithm may be able to maintain high CSRs in the presence of overlapping spikes.

In addition to the simulator developed in [18], the spike trains in the wave_clus database are also considered in the experiments. Table 10 shows the resulting comparisons. Similar to the results shown in Table 7, Table 8 and Table 9, we can also see from Table 10 that the CSR performances of the PCA, GHA and the proposed algorithm are comparable. In addition, the proposed algorithm outperforms the zero-crossing algorithm. Although PCA and GHA offer higher classification accuracy, the algorithms have higher computational complexities. We can see from Table 6 that their hardware implementations may have large area costs and power consumption. The computational complexities of zero-crossing are low. However, the algorithm has inferior CSR values. In particular, for the spike train data “C_Difficult2_noise005” in Table 10, the CSR of the zero-crossing is only 69.92%. By contrast, the proposed algorithm still maintain high accuracy (i.e., 82.13%) for the data. Therefore, the proposed algorithms have the advantages of low computational complexities for efficient hardware implementation and are capable of providing feature vectors for spike classification with high accuracy.

## 5. Conclusions

We have implemented the proposed architecture for spike sorting by ASIC with 90-nm technology. Experimental results reveal that the proposed architecture has the advantages of low area costs, low power consumption and high classification accuracy. For the 32-channel design example provided in the paper, the normalized area is 21,127 μm${}^{2}$/channel, which is the lowest as compared with the existing designs considered in the paper. When operating at 1 MHz, the proposed architecture consumes normalized power of 20.03 μW/channel. The CSR values of the FCM based on the feature vectors provided by the proposed algorithm are also comparable to those of the PCA and GHA techniques. The proposed architecture therefore is an effective alternative to the applications where implantable spike sorting circuits with low power consumption, low area costs and high CSR are desired.

## Figures and Tables

**Table 1:** The area complexities of the proposed feature extraction circuit.

	Comparators	Adders/Subtractors	Multipliers/Dividers	Registers	Multiplexers
GMS Unit	O(1)	0	0	O(1)	O(1)
ACC Unit	0	O(1)	0	O(1)	O(1)
GMS Merger Unit	O(1)	0	0	0	O(1)
ACC Merger Unit	0	O(1)	0	0	O(1)
FS Unit	0	O(1)	O(1)	0	0
Total Leave Nodes	O(K)	O(K)	0	O(K)	O(K)
Total Intermediate Nodes	O(K)	O(K)	0	0	O(K)
Total Root Nodes	0	O(1)	O(1)	0	0
Total	O(K)	O(K)	O(1)	O(K)	O(K)

**Table 2:** The area complexities of the proposed spike sorting circuit.

	Comparators	Adders/Subtractors	Multipliers/Dividers	Registers	Multiplexers
NEO Circuit	O(1)	O(1)	O(1)	O(MN)	0
Spike Buffer	0	0	0	O(MN)	O(1)
Feature Extraction	O(K)	O(K)	O(1)	O(K)	O(K)
Total	O(K)	O(K)	O(1)	O(MN+K)	O(K)

**Table 3:** The area (μm${}^{2}$) of the proposed circuit for different numbers of channels *M* and segments *K*.

Number of Segments *K*	Number of Channels *M*					
	2	4	8	16	32	64
1	58,008	98,990	180,665	346,647	676,085	1,337,493
2	65,625	106,607	188,282	354,264	683,702	1,345,110
4	80,859	121,841	203,516	369,498	698,936	1,360,344

**Table 4:** The normalized area (μm${}^{2}$/channel) of the proposed circuit for different numbers of channels *M* and segments *K*.

Number of Segments *K*	Number of Channels *M*					
	2	4	8	16	32	64
1	29,004	24,747	22,583	21,665	21,127	20,898
2	32,813	26,652	23,535	22,142	21,366	21,017
4	40,430	30,460	25,440	23,094	21,842	21,255

**Table 5:** The normalized power consumption (μW/channel) of the proposed circuit with and without clock gating for different numbers of channels *M*.

	Number of Channels *M*					
	2	4	8	16	32	64
No Clock Gating	43.08	36.55	33.00	31.90	31.10	30.69
Clock Gating	32.98	26.58	23.13	21.69	20.03	20.53
Power Reduction	23%	27%	29%	32%	36%	33%

**Table 6:** The comparisons of various spike sorting architectures.

Architecture	Normalized	Normalized	Clock	# of	Spike	Technology	Detection	Feature
	Area	Power	Rate	Channels	Dimension			Extraction
[8]	255,495 μm${}^{2}$/ch.	521 μW/ch.	1 MHz	1	64	90 nm	No	PCA
[9]	1,770,000 μm${}^{2}$/ch.	256.875 μW/ch.	N/A	16	N/A	350 nm	NEO	PCA
[10]	268,000 μm${}^{2}$/ch.	8.589 μW/ch.	281.25 KHz	1	64	130 nm	No	SPIRIT
[11]	83,159 μm${}^{2}$/ch.	78.719 μW/ch.	1 MHz	32	64	90 nm	NEO	GHA
Proposed	21,127 μm${}^{2}$/ch.	20.03 μW/ch.	1 MHz	32	64	90 nm	NEO	Proposed

**Table 7:** The CSRs of the various feature extraction algorithms for different SNR levels. The spike source data are obtained by the simulator in [18].

Algorithms	SNR (dB)			
	1	4	6	8
PCA [8]	99.57%	99.70%	99.64%	99.82%
GHA [11]	99.30%	99.58%	99.82%	99.82%
Zero-Crossing [15]	96.32%	96.71%	95.44%	95.57%
Proposed	96.47%	97.22%	97.13%	97.52%

**Table 8:** The CSRs of the various feature extraction algorithms for different number of interfering neurons. The spike source data are obtained by the simulator in [18].

Algorithms	Number of Interfering Neurons			
	0	2	4	6
PCA [8]	99.70%	99.68%	99.76%	99.58%
GHA [11]	99.58%	99.66%	99.58%	99.51%
Proposed	97.22%	96.09%	95.56%	95.44%

**Table 9:** The CSRs of the various feature extraction algorithms for different percentages of overlapping spikes. The spike source data are obtained by the simulator in [18].

Algorithms	Percentage of Overlapping Spikes			
	15%	20%	30%	40%
PCA [8]	99.80%	99.82%	99.75%	99.35%
GHA [11]	99.76%	99.82%	99.75%	99.36%
Proposed	98.06%	97.52%	96.25%	94.84%

**Table 10:** The CSRs of the FCM algorithm by clustering the feature vectors produced by various feature extraction algorithms. The spike source data are obtained from the wave_clus database [20].

Algorithms	File Names			
	C_Easy1_Noise01	C_Easy2_Noise01	C_Easy2_Noise005	C_Difficult2_Noise005
PCA [8]	99.32%	96.68%	98.45%	98.57%
GHA [11]	99.32%	94.35%	98.15%	81.66%
Zero-Crossing [15]	89.61%	79.86%	92.26%	69.92%
Proposed	93.00%	90.57%	93.90%	82.13%

## Abbreviations

The following abbreviations are used in this manuscript:

ASIC	Application-Specific Integrated Circuit
CSR	Classification Success Rate
FCM	Fuzzy C-Means
FPGA	Field Programmable Gate Array
GHA	Generalized Hebbian Algorithm
GMS	Global Minimum and maximum Search
NEO	Non-linear Energy Operator
PCA	Principal Component Analysis
FS	Feature Search
ACC	ACCumulation
